# How policymakers and other leaders can build a more sustainable post-COVID-19 ‘normal’

**DOI:** 10.1007/s43621-022-00074-x

**Published:** 2022-02-17

**Authors:** Peter Bragge, Ursula Becker, Thomas Breu, Henrik Carlsen, David Griggs, John N. Lavis, Caroline Zimm, Anne-Sophie Stevance

**Affiliations:** 1 Monash Sustainable Development Institute, Monash University, Melbourne, Australia; 2 Deutsche Gesellschaft Fuer Internationale Zusammenarbeit (GIZ) GmbH, Bonn, Germany; 3 Centre for Development and Environment (CDE), University of Bern, Bern, Switzerland; 4 Stockholm Environment Institute, Stockholm, Sweden; 5 McMaster Health Forum, McMaster University, Hamilton, Canada; 6 International Institute for Applied Systems Analysis, Laxenburg, Austria; 7 International Science Council, Paris, France

## Introduction

The UN 2030 Agenda’s 17 Sustainable Development Goals (SDGs) and the COVID-19 pandemic share two important characteristics. They are global challenges that if not met, pose risks to all citizens. Furthermore, responses need to be system-level, rather than sectoral. COVID-19 has illuminated three complementary, compelling actions that can address these challenges—work across silos; visibly use science in policy; and harness simultaneous global interruption to habits. This commentary describes these using worked examples and suggests actions for policymakers and other leaders. Acknowledging that the full SDG agenda is of much broader multidimensional scope than the COVID-19 pandemic, the SDG examples focus on environmental sustainability.

### Work across silos

To manage the pandemic through public health responses such as physical distancing, policymakers had to pivot from ‘silo’-based approaches to coordinating a collective response across multiple fields including science, business, health, social sciences and technology [1–3]. Each SDG goal within the 2030 Agenda also requires transdisciplinary research—spanning disciplinary and sectoral boundaries while integrating diverse academic and non-academic knowledge [4–7]. Science focussing on SDG interactions—which identifies, analyse and explores synergies (mutual benefits) and trade-offs (action in one area compromising progress in another) between SDG targets when addressing global systemic challenges—is a key response to the need for transdisciplinary ‘silo-breaking’ [8–11]. This involves various actors including policymakers, practitioners, citizens and researchers - considering how policies and practices to address one SDG may impact (positively or negatively) upon progress in another. For example, efforts to increase use of renewable energy (SDG target 7.2) also reduce air pollution (SDG target 3.9); conversely, agricultural practices to produce food achieve health targets (SDG 3) but agricultural practices (for example land clearing) (SDG 2) can negatively damage ecosystems (SDG 15) [8]. Therefore, although actors may share a commitment to sustainability, each will bring their specific organisational and/or sectoral interests to the table. Navigating the various conflicts that can arise is pivotal to meaningful outcomes of transdisciplinary dialogue [12].

### Work across silos: examples

Green recovery measures for energy, transport, buildings, industry and agriculture, have great ‘silo-breaking’ potential, harnessing synergies between economic stimulus and pressing environmental and social challenges [13]. Green recovery supports achieving the SDGs and the Paris Agreement through dedicated budgetary commitments considering such interlinkages. Prior to the pandemic, over 20 countries had already committed to linking their budgets to the SDGs [14]. The pandemic led to over 300 fiscal rescue measures to sustain economies with an emphasis on maintaining the status quo, for example through business and worker compensation [15, 16]. The challenge lies in achieving a ‘green’ recovery of the status-quo by ensuring that these investments are directed towards transition to a net zero greenhouse gas (GHG) emission economy. Although the proportion of ‘green’ spending grew throughout 2020 there is room for improvement to leverage the transformative potential of public spending. While the total green spending announced in 2020 was USD697bn, it is only a fraction of total *overall annual* spending; just 23.4% of *overall* ‘recovery’ spending and a mere 4.2% of *overall* announced spending globally is likely to reduce GHG emissions [13].

### Work across silos: suggested actions

Now, with existing and new vulnerabilities brought into view by the pandemic, is the moment to embed governance and transdisciplinary research as new ‘business as usual’ for dealing with complex challenges. Actions across technology, business, social, health and science domains require coordination to achieve policy coherence. Coordination functions include facilitating shared understanding of problems and solutions—for example through joint committees and silo crossover innovation programs [17]; optimising policy and institutional coherence through consideration of interactions and budgeting processes [18]; and tying stimulus measures to green commitments [1, 13]. This echoes and builds upon pre-pandemic calls for a ‘governance by goals’ SDG approach characterised by flexible, responsive governance and close collaboration between institutions and transdisciplinary SDG researchers [17, 19]. Such an approach could operate at all levels of government within a country or (ideally) illustrate more universal principles that could be adapted to specific contexts and settings. This is a challenging proposition carrying substantial logistical, intellectual and social complexities. Understanding and application of the science of policy integration is hampered by varying terminologies; cynicism with politics threatens to reduce buy-in to the entire enterprise; and evidence about precisely how ‘joined-up’ government works and how its purported benefits can be measured is lacking [20–22].

However there are two compelling reasons for investing in this effort. First, although there will always be a need for monodisciplinary and portfolio-specific knowledge and action to address focused challenges in defined areas, this is insufficient to address the global SDG and COVID challenges. Second, there is less than ten years to achieve the SDGs and both government and research sectors are disrupted by the COVID-19 pandemic. Therefore, potential return on investment in transdisciplinary approaches outweighs that for returning to pre-pandemic silos.

### Visibly use science in policy

COVID-19 has made science and its impact on communities visible. Political leaders regularly stand side-by-side with scientists delivering COVID-19 information and promoting public health messaging [23, 24]. Terms such as ‘flattening the curve’ became everyday language in 2020 and science-based public health measures including physical distancing and mask-wearing continue to impact day-to-day living [23, 25].

### Visibly use science in policy: examples

COVID-19 has placed a spotlight on how citizens understand and engage with science. Science literacy—understanding probability, risks and cost–benefit—is required to understand COVID-19 data and the rationale behind public health measures [23]. Mobile collection of personal location data and COVID-19 status to map the virus has also enhanced awareness of the importance of citizen science [24]. In parallel, demand for real-time scientific support of decision-making has increased dramatically [26, 27]. Building on at least two decades of evidence-informed policy science [28, 29], the evidence community has responded to this demand through initiatives such as the COVID-19 Evidence Network to support Decision-making (COVID-END)—an international research synthesis, policy and public health effort to systematically identify, appraise and map best-available COVID-19 research syntheses to a broad array of COVID-19 response and recovery decisions [27, 30, 31]. This culminated in a recently-released report documenting recommendations for improving use of science in policy and practice [32]. Perhaps the most powerful recent example of visible connection of SDG science to policy is the ‘Code Red for Humanity’ report of the Intergovernmental Panel on Climate Change (IPPC) [33] which provided an alarming backdrop to the Glasgow COP26 Summit in late 2021.

### Visibly use science in policy: suggested actions

High visibility of science-informed policy and calls for greater application of science-based policy to both COVID-19 and sustainability [34, 35] create arguably the most favourable conditions yet in which to elevate the role of science [36, 37]. Knowledge produced by scientists and other professions (for example business, logistics, manufacturing) and the perspectives of citizens representing diverse groups in society—need to be represented at government and other decision-making tables. Pivoting COVID-19 crisis management panels to a broader sustainable COVID-19 recovery remit and remaining next to ministers when announcing priorities and actions can keep scientific and other knowledge visible in public policy in the long term in support of realisation of the 2030 Agenda, in doing so continuing to engage the wider community in the importance of harnessing such knowledge and insights to drive effective and meaningful action. An informed and engaged citizenry can empower politicians to pursue system-wide transformations [38]. Restrictions in travel and working from home have enhanced appreciation of green and blue spaces, suggesting enhanced public support for sustainable living programs [39].

The research community must respond with the best available scientific knowledge. This requires long-term global investment to ensure sustainability of successful COVID-19 research responses such as creation of transdisciplinary networks, practitioners who can support science-informed policy and open data sharing. We must also learn from what has gone wrong. Insatiable appetite for science (perhaps combined with skewed incentives to meet academic publishing KPIs) fuelled acceptance of poor-quality evidence; poor organisation of clinical research resulted in numerous underpowered, wasteful trials; and evidence reviews were similarly disorganised, duplicative and low-quality [27]. These deficiencies must be urgently addressed so that science can deliver on its promise to support effective and sustainable COVID-19 recovery and deliver on the 2030 Agenda.

### Harness simultaneous global interruption to habits

Habits are behaviours conducted frequently and usually at the same location and time with minimal conscious intent, preserving cognitive effort for more complex tasks [40]. The automatic nature of habits also makes them very hard to break. ‘Habit discontinuity’—disruption due to life changes such as moving house or having a baby—has been shown to be effective in breaking old or embedding new habits [40]. Ordinarily, such life chances are rare and asynchronous across populations. Widespread, population-wide disruption to work, social and other routines due to COVID-19 [41] is therefore an extremely rare window of opportunity to embed SDG-friendly and healthy COVID-19 recovery habits globally.

### Harness simultaneous global interruption to habits: examples

There are numerous examples of the effect of COVID-19 on habits. First, some habits—for example handshakes, standing close to people and touching our face—have been disrupted by public health measures [42]. Second, lifestyle changes have created new healthy habits. Reallocation of street space to pedestrians and cyclists has broken barriers to active travel, reducing emissions and enhancing health [43]; the need to address mental wellbeing has encouraged people to implement strategies to improve sleep, increase exercise and manage difficult thoughts [44, 45]. Finally, there are negative aspects of habit disruption including quarantine-related weight gain, depression and increases in domestic violence [46, 47]; and disproportionate impact of austerity and containment measures on disadvantaged groups, for example due to increased energy bills and exposure to food poverty [48]—effects which are more widespread in low- and middle-income countries with less access to government safety nets [49].

### Harness simultaneous global interruption to habits: suggested actions

Clearly, embedding positive and mitigating negative outcomes is the key to optimising pandemic-imposed habit discontinuity. Positive reinforcement should include and move beyond climate change, which is already on the sustainable COVID-19 recovery agenda [50, 51]—albeit with variable alignment between rhetoric and action at a country level [52]. Fortunately, positive habits by definition, are self-sustaining. The habit of commuting by bike persists even when trips become complex [53] (which is possible if changes to road infrastructure to accommodate active transport are temporary); and enhanced interest in urban nature has positive flow on effects to well-being, social contact, and connection to the outside world [39]. However, the ‘stickiness’ of habits is a double-edged sword—it works against the breaking of bad habits such as weight gain, which has been shown to be associated with mental stresses arising from COVID-19 [46].

Policymakers can reinforce positive and reduce negative habits in several ways—first, by reducing ‘friction’ associated with sustainable habits (for example by retaining active transport lanes on roads) and increasing friction for less sustainable alternatives (traffic zones, reduced speed limits for cars); second, by harnessing context-specific cues (recycling signs, reminders); and finally, through (dis-)incentives (plastic bag charges, smart energy meters) [53]. System-level considerations include strengthening of social safety nets to address widening inequalities resulting from COVID-19 and consideration of how global habit change can contribute to global reduction of poverty and food insecurity. Policy, industry and other leadership is critical to embedding and enabling positive habits; without it, the ‘thinking (and living) out of the box’ that arose during lockdown will be temporary. To enhance visibility of these opportunities to both leaders and citizens, major reported habit-changes arising from COVID-19 could be mapped to relevant SDG targets, with results shared in lay language (e.g. ‘the top 10 new habits to keep for a better world’).

## Conclusion

Solving interdependent, complex global sustainability challenges through the implementation of the 2030 Agenda and its 17 associated SDGs will define this century. The COVID-19 pandemic has shown the frightening susceptibility of all societies to global crises and a disturbing, relatable glimpse of what such crises can bring. This commentary has focused on three actions that can assist transition from COVID-19 response to sustainable COVID-19 recovery. The window for these actions will not remain open for long; they must be seized by humanity—science, policy and society at large. If this work starts now, we can frame sustainability efforts beyond COVID-19 recovery and 2030 to the equally important long-term 2050 and 2100 horizons for long-term planetary resilience.

*Literature cited in this commentary was drawn from the Social Systems Evidence (SSE) database (socialsystemsevidence.org) which is co-led by report authors Peter Bragge and John Lavis. SSE is the largest database of review-level evidence mapped to the Sustainable Development Goals in the world. The three actions were developed through a series of meetings of the author team convened by the International Science Council’s SDG Interactions Working Group. Group members identified further literature and worked examples through their involvement in sustainable development research at their respective organisations which in addition to ISC were Monash Sustainable Development Institute; Deutsche Gesellschaft fuer Internationale Zusammenarbeit (GIZ) GmbH; Centre for Development and Environment (CDE), University of Bern; McMaster Health Forum, McMaster University and Africa Centre for Evidence, University of Johannesburg; and the International Institute for Applied Systems Analysis.*
